# Supplementary material for: Measuring error rates in genomic perturbation screens: gold standards for human functional genomics
Source: Mol Syst Biol. 2014 Jul 1;10(7):733. doi: 10.15252/msb.20145216 (PMC4299491; doi:10.15252/msb.20145216)
Supplement: Supplementary file 7 — Supplementary Figure S1 [file msb0010-0733-sd7.pdf]

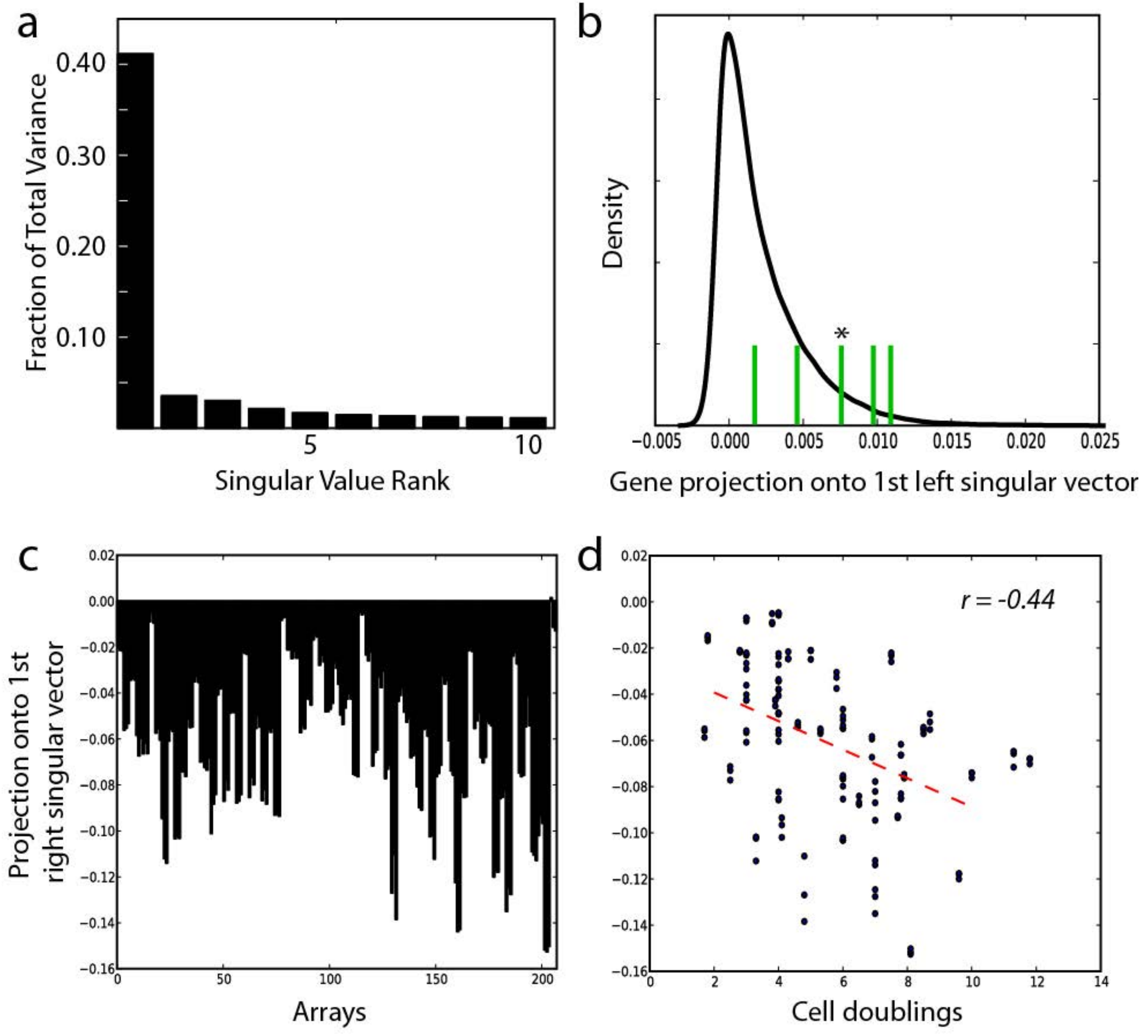
a
Fraction of Total Variance
0.40
0.30
0.20
0.10
5
10
Singular Value Rank
b
Density
*
−0.005
0.000
0.005
0.010
0.015
0.020
0.025
Gene projection onto 1st left singular vector
c
Projection onto 1st right singular vector
0.02
0.00
−0.02
−0.04
−0.06
−0.08
−0.10
−0.12
−0.14
−0.16
0
50
100
150
200
Arrays
d
r = -0.44
0.02
0.00
−0.02
−0.04
−0.06
−0.08
−0.10
−0.12
−0.14
−0.16
0
2
4
6
8
10
12
14
Cell doublings

Figure S1. Singular value decomposition of the raw shRNA fold change matrix.

(a) Fraction of total variance explained by the top 10 singular values by SVD of the first half of the fold change matrix. Signal is concentrated in the first vector, which accounts for ~42% of total variance.

(b) The distribution of all hairpin projections onto the first left singular vector (black). Strong positive projections indicate dropout across informative samples. Green, example of hairpins targeting a given gene. Value of the projection of the median hairpin (starred) is used for calculating P-value, as described in the main text and methods.

(c) Projection of individual samples onto first right singular vector. Strong negative projections indicate more informative screens.

(d) Sample projection onto first right singular vector is correlated with the number of cell doublings at which the sample is measured; more cell doublings yields more informative screens.
