# Supplementary material for: Measuring error rates in genomic perturbation screens: gold standards for human functional genomics
Source: Mol Syst Biol. 2014 Jul 1;10(7):733. doi: 10.15252/msb.20145216 (PMC4299491; doi:10.15252/msb.20145216)
Supplement: Supplementary file 8 — Supplementary Figure S2 [file msb0010-0733-sd8.pdf]

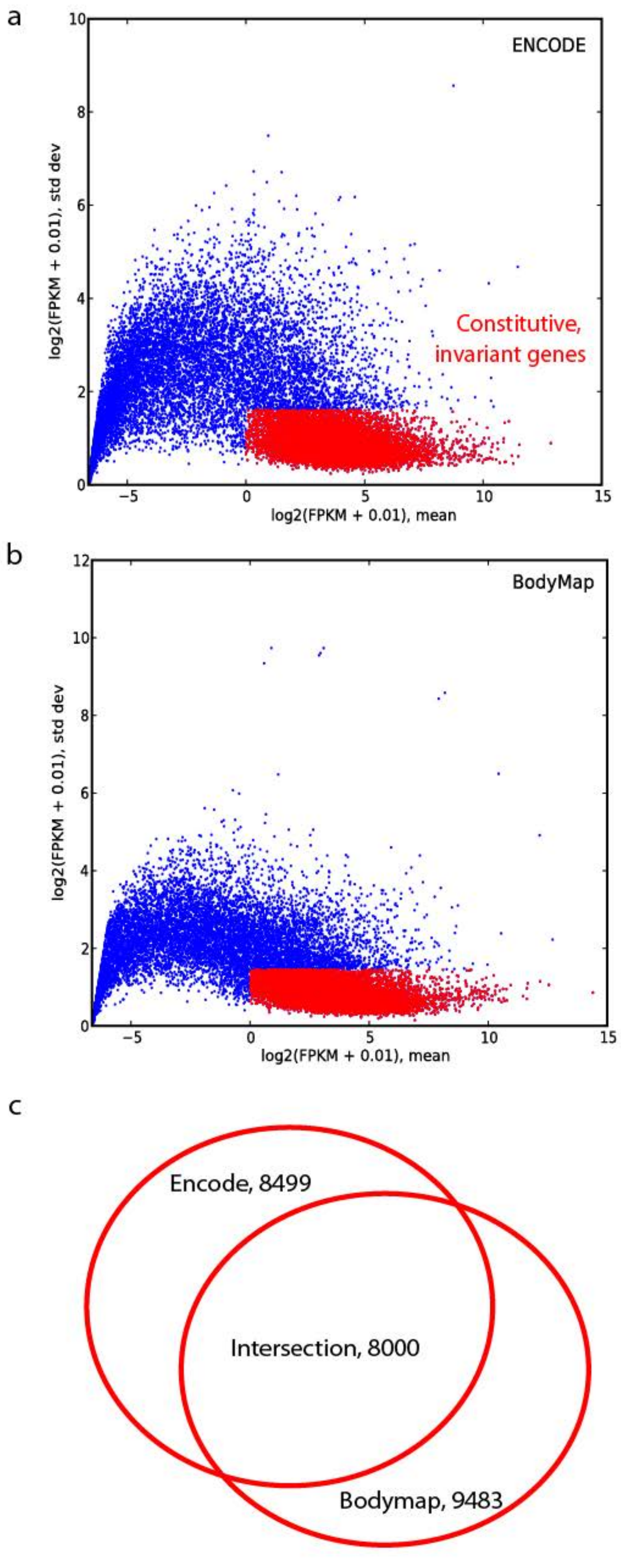
a
ENCODE
Constitutive, invariant genes
log2(FPKM + 0.01), std dev
log2(FPKM + 0.01), mean
b
BodyMap
log2(FPKM + 0.01), std dev
log2(FPKM + 0.01), mean
c
Encode, 8499
Intersection, 8000
Bodymap, 9483

Figure S2. Constitutive, invariant genes. (a) Mean vs. standard deviation of log(FPKM + 0.01) across 17 ENCODE cell line samples. Red indicates genes with constitutive (expression > 0), invariant (stdev $<$ mean(stedev)) expression. (b) Same plot for Illumina BodyMap compendium of RNA-seq data from 16 healthy human tissues. (c) Venn diagram indicating intersection of constitutive, invariant genes across the two data sets.
