# Supplementary material for: Measuring error rates in genomic perturbation screens: gold standards for human functional genomics
Source: Mol Syst Biol. 2014 Jul 1;10(7):733. doi: 10.15252/msb.20145216 (PMC4299491; doi:10.15252/msb.20145216)
Supplement: Supplementary file 9 — Supplementary Figure S3 [file msb0010-0733-sd9.pdf]

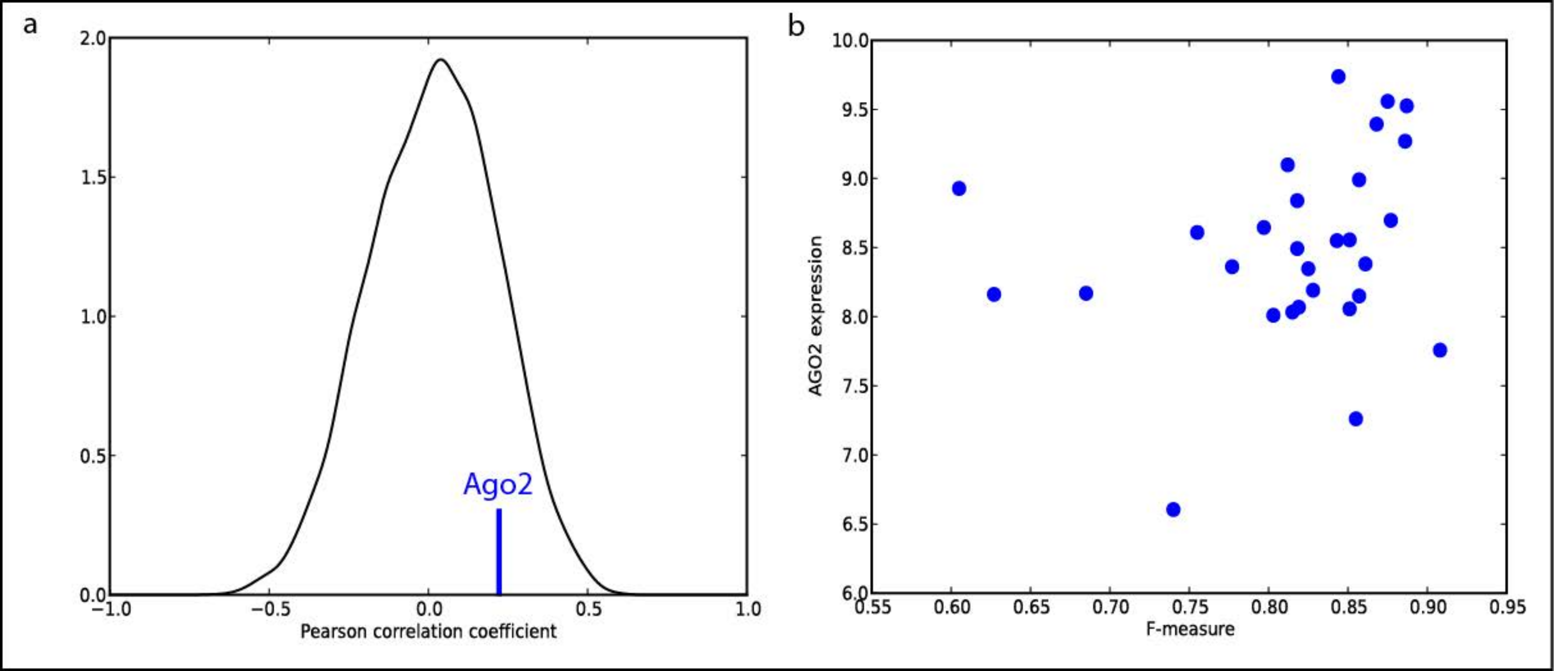
a
b
Ago2
Pearson correlation coefficient
F-measure
AGO2 expression
2.0
1.5
1.0
0.5
0.0
−1.0
−0.5
0.0
0.5
1.0
10.0
9.5
9.0
8.5
8.0
7.5
7.0
6.5
6.0
0.55
0.60
0.65
0.70
0.75
0.80
0.85
0.90
0.95

Figure S3. Expression vs. screen F-measure across 29 BRCA cell lines.

(a) Distribution of correlations across all expressed genes. Ago2 correlation =0.22.

(b) Scatterplot of F-measure vs. Ago2 expression across 29 BRCA cell lines.
