# Supplementary material for: Measuring error rates in genomic perturbation screens: gold standards for human functional genomics
Source: Mol Syst Biol. 2014 Jul 1;10(7):733. doi: 10.15252/msb.20145216 (PMC4299491; doi:10.15252/msb.20145216)
Supplement: Supplementary file 10 — Supplementary Figure S4 [file msb0010-0733-sd10.pdf]

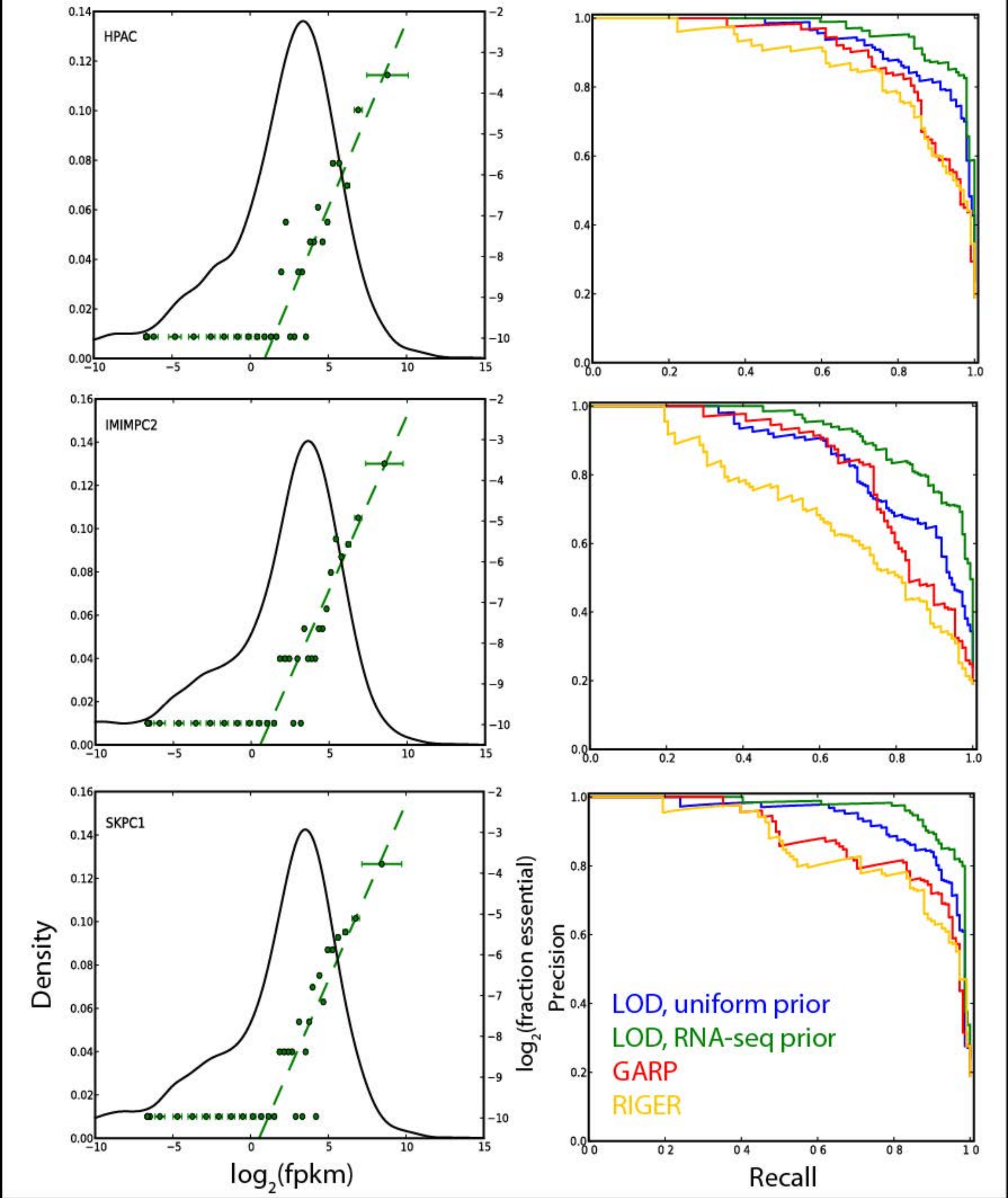

HPAC
IMIMPC2
SKPC1
Density
log2(fpkm)
log2(fraction essential)
Precision
Recall
LOD, uniform prior
LOD, RNA-seq prior
GARP
RIGER

Figure S4. Integrating gene expression into the Bayesian classifier.

(left column) For RNAi screens with matched gene expression data, genes are binned by expression level and the fraction of reference essentials in each bin (right Y axis) is plotted against the mean expression of genes in the bin (green points). A linear fit on the log-log plot (green dashed line) is integrated into the Bayesian classifier as an informative prior.

(right column) Integrating expression data improves the performance of the classifier (green) over the base algorithm (blue). Both forms show better performance than other algorithms such as GARP (red) and RIGER (gold).
