# Supplementary material for: Measuring error rates in genomic perturbation screens: gold standards for human functional genomics
Source: Mol Syst Biol. 2014 Jul 1;10(7):733. doi: 10.15252/msb.20145216 (PMC4299491; doi:10.15252/msb.20145216)
Supplement: Supplementary file 11 — Supplementary Figure S5 [file msb0010-0733-sd11.pdf]

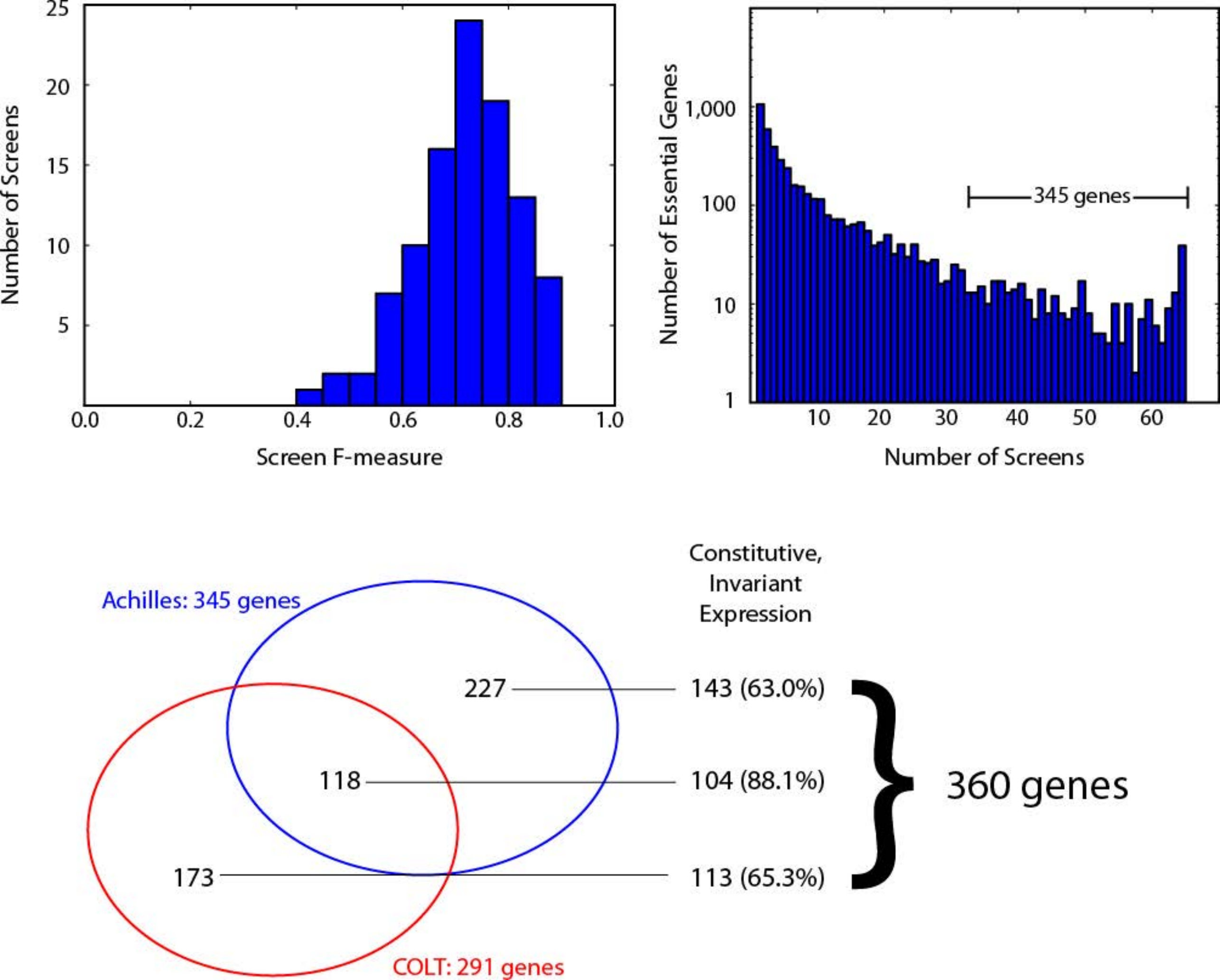

Number of Screens
Screen F-measure
Number of Essential Genes
Number of Screens
345 genes
Achilles: 345 genes
COLT: 291 genes
Constitutive, Invariant Expression
227
118
173
143 (63.0%)
104 (88.1%)
113 (65.3%)
360 genes

Figure S5. Core constitutive essentials from Project Achilles data.

(top left) Distribution of F-measures of 102 Achilles screens. Screens with F>0.7 were retained for further analysis (n=65).

(Top right) Histogram of number of screens in which each gene is classified as essential (BF>5; average FDR ~16%) across the 65 screens. Genes essential in 33 of 65 screens (n=345) are classified as core essentials.

(Bottom) Venn diagram comparing Achilles and Colt core essentials. Each set contains roughly the same proportion of genes that meet our criteria for constitutive, invariant mRNA expression. The union of these filtered genes is our integrated set of core essential genes (n=360).
